# Inhibitors compounds of the flavivirus replication process

**DOI:** 10.1186/s12985-017-0761-1

**Published:** 2017-05-15

**Authors:** Leidy L. García, Leonardo Padilla, Jhon C. Castaño

**Affiliations:** Group of Molecular Immunology, Universidad del Quindío, Armenia (Quindío), Colombia

**Keywords:** Flavivirus, NS5 protein, Inhibitors

## Abstract

Flaviviruses are small viruses with single-stranded RNA, which include the yellow fever virus, dengue virus, West Nile virus, Japanese encephalitis virus, tick-borne encephalitis virus, and Zika virus; and are causal agents of the most important emerging diseases that have no available treatment to date. In recent years, the strategy has focused on the development of replication inhibitors of these viruses designed to act mainly by affecting the activity of enzyme proteins, such as NS3 and NS5, which perform important functions in the viral replication process. This article describes the importance of flaviviruses and the development of molecules used as inhibitors of viral replication in this genus.

## Background

Viruses of the *Flaviviridae* family are small enveloped viruses (~50 nm) [1] with a single-stranded RNA genome of positive polarity. The complete genome is 9500-12,500 nucleotides long and codes for a precursor polyprotein processed by viral and cellular proteases into structural proteins involved in capsid construction, as well as nonstructural proteins implicated in virus replication [2].

**Table 1:** Main compounds used as flavivirus inhibitors

Protein	Compound	Flavivirus	References	State
E	NITD-448	DENV	Lim et al., 2013a [38]	NITD-448 inhibited E protein-mediated membrane fusión as well as DENV-2 infection. Due to the large molecular weight of this compound, por selectivity, and pharmacokinetic properties, this compound series was not further pursued.
	Compound 6	DENV	Lim et al., 2013a [38]	Compound 6 has low potency and high plasma protein-binding activity due to lipophilicity that prevented further development of the compound.
	P02	DENV, YFV	Zhou et al., 2008 [44], Lim et al., 2013a [38]	P02 inhibits viral replication at micromolar concentrations. No follow-up studies have been reported for this compound.
	D02, D04 and D05	DENV	Zhou et al.,2008 [44]	D02, D04 and D05 inhibit the virus life cycle. This compounds reduced viral replication activity.
	A5	DENV, YFV, WNV	Lim et al., 2013a [38]	A5 has low micromolar activity against DENV, WNV, and YFV. No follow-up studies have been reported for this compound.
	1662G07	DENV	Lim et al., 2013a [38]	SAR studies led to analogs with activity against DENV-2, some activity against DENV-4, but weak activity against DENV-1 and −3. The antiviral spectrum of this compound needs to be evaluated.
	Castanosper-mine	DENV	Lim et al., 2013a [38]	Castanospermine inhibit DENV replication, disrupting folding of DENV structural proteins prM and E, as well as NS1.
	Celgosivir	DENV	Lim et al., 2013a [38]	Celgosivir inhibit DENV replication, disrupting folding of DENV structural proteins prM and E, as well as NS1. A phase Ib clinical trial is currently ongoing to evaluate the activity, pharmacokinetics, safety, and tolerability of Celgosivir in dengue patients.
	Peptide DN59	DENV	Lok et al., 2012; [46]﻿ Badani et al., 2014 [45]	Peptide DN59 inhibits the infectivity of flaviviruses by releasing their genome.
C	ST-148	DENV	Lim et al., 2013a [38]	ST-148 has limited oral bioavailability. Greater preclinical development is warranted.
NS3- ProteasE	Bowman-Birk inhibitor	DENV	Bollati et al., 2010 [2]	A starting point is provided for the design of specific inhibitors. The current situation of this compounds is unknown.
	BP13944	DENV	Yang et al.,2014 [48]	BP13944 inhibited viral replication or RNA synthesis in all four DENV serotypes. Further work is required to determine the interaction mechanism.
	Compound 32 (keto amides)	DENV	Steuer et al., 2011 [49]	An inhibitory effect on DENV replication was determined in a dose-dependent manner. Cytotoxicity in cell culture is unknown.
	Peptide WCW-NH2	DENV	Prusis et al., 2013 [50]	Peptide WCW-NH2 inhibited protease in the four serotypes.
	ARDP0006 and ARDP0009	DENV	Tonlimson and Watowich, 2011 [51]	ARDP0006 and ARDP0009 inhibited DENV-2 virus replication in cell culture. Promising compounds for future research.
	Aprotinin	DENV	Noble et al., 2010 [53]	Aprotinin envelops the enzyme and prevents the substrate from accessing the protease active site.
NS3- HelicasE	Halogenated benztrioles	WNV	Sampath and Padmanabhan 2009 [3]	Halogenated benztrioles were good, and selective inhibitor of the West Nile virus.
	Ivermectin	DENV, JEV, YFV	Mastrangelo et al., 2012 [55]; Sweeney et al., 2015 [57]	Promising compound as the first specific therapy against flaviviruses (patent application EP2010/065880).
	ST-610	DENV	Lim et al., 2013a [38]; Sweeney et al., 2015 [57]	ST610 potently and selectively inhibited all four DENV serotypes in cell culture. For future trials, the pharmacokinetic properties of this compound should be improved.
	Suramin	DENV	Basavannacharya, Vasudeban, 2014 [58]; Sweeney et al., 2015 [57]	Suramin acted as a potent NS3 helicase inhibitor of dengue virus by a non-competitive mode of inhibition.
	Analogues ML283	DENV	Sweeney et al., 2015 [57]	Analogues ML28331 were potent inhibitors of DENV NS3- catalyzed ATP hydrolysis. These trials serve as a tool to find more inhibitory compounds.
	Pyrrolone	DENV, WNV	Sweeney et al., 2015 [57]	Pyrrolone inhibited DENV replicon and WNV replication in cell culture. These trials serve as a tool to find more inhibitory compounds.
NS4 B	SDM25N	DENV	Van Cleef et al., 2013 [59]	SDM25N showed antiviral activity against wild-type DENV2 in both Hela and BHK-21 cells, but not in the C6/36 cell line. Further studies are required.
	NITD-618	DENV, WNV, YFV	Van Cleef et al., 2013 [59]; Lim et al., 2013a [38]	The high lipophilicity of NITD-618 resulted in poor pharmacokinetic properties which hindered testing its activity against DENV in AG129 mice. It was inactive against WNV and YFV.
NS5-metHYl-transfe-rasE	Sinefungin	DENV, WNV	Lim et al., 2015 [61]	The affinity for Sinefungin is approximately six times higher than for SAM. Sinefungin is non-cell permeable. This compound does not show progress
	S-adenosyl homocysteine	DENV	Lim et al., 2015 [61]	S-adenosyl homocysteine is non-cell permeable. This compound does not show progress.
	Compound 10	DENV	Lim et al., 2013a [38] ﻿ Lim et al., 2015 [61]	Compound 10 is non-cell permeable. This compound does not show progress.
	GMP	DENV	Lim et al.,2015 [61]	GMP is non-cell permeable. This compound does not show progress.
	BG-323	DENV	Lim et al., 2015 [61]	BG-323 showed in vitro inhibition of DENV. The current situation of this compound is unknown.
	Aurintricarboxylic acid	DENV, YFV	Lim et al., 2015 [61]	Aurintricarboxylic acid inhibited the DENV MTase and YFV. The current situation of this compound is unknown.
	4-HPR	DENV	Lim et al. 2015 [61]; Lai et al., 2017 [10]	4-HPR showed efficacy in DENV mouse models. Promising compound. Due to its tolerable human profile, it could be a treatment for patients with DENV.
	Ivermectin	DENV	Lim et al., 2013a [38]; Lim et al., 2015 [61]; Lai et al., 2017 [10]	Ivermectin is a promising compound. The estimated study completion date was February 2016 (ClinicalTrials.gov identifier: NCT02045069).
	Ribavirin	DENV	Lim et al., 2013a [38]	Ribavirin has limited use due to its toxicity, thus, decreasing its clinical effectiveness.
NS5-polYmera-sE	N-(4 hydroxy-phenyl) retinamide	DENV	Lim et al., 2015 [61]	N-(4-hydroxyphenyl) retinamide did not affect NS5 RdRp activity, but inhibited DENV replication in cells.
	Ivermectin	DENV	Tay et al., 2013 [56]; Lim et al., 2015 [61]	Ivermectin did not affect NS5 RdRp activity, but inhibited DENV replication in cells. However, problems related to the toxicity of ivermectin may present significant challenges for its potential use in anti-DENV therapy.
	7-deaza-2’-C methyladeno-sine (7DMA)	ZIKV	Zmurko et al., 2016 [67]	7DMA was shown to reduce viremia and delay the time to disease progression in virus-infected mice.
	3’dGTP	DENV	Malet et al., 2008 [8]	3’dGTP showed low micromolar IC50 values in in vitro DENV2 RdRp activity tests using a poly(rC) template.
	ddGTP	DENV	Malet et al., 2008 [8]	ddGTP showed low micromolar IC50 values in in vitro DENV2 RdRp activity tests using a poly(rC) template.
	3’dioxolano 3’dGTP	DENV	Malet et al., 2008 [8]	3’dioxolano 3’dGTP showed low micromolar IC50 values in in vitro DENV2 RdRp activity tests using a poly(rC) template.
	2’-O-metil GTP	DENV	Malet et al., 2008 [8]	2’-O-metil GTP showed low micromolar IC50 values in in vitro DENV2 RdRp activity tests using a poly(rC) template.
	HPA 23	DENV	Malet et al., 2008 [8]	HPA 23 Inhibited virus replication in Vero cells. The current situation of this compound is unknown.
	NITD 008	DENV, WNV, YFV	Lim et al., 2013a [38]; Caillet et al., 2014 [30]	Severe side-effects were observed in rats and dogs. These results led to the termination of NITD-008 for further development for DENV treatment.
	NITD 203	DENV, WNV, YFV	Calleit et al., 2014 [30]	NITD 203 showed in vivo toxicity after 2 weeks of administration in rats and dogs.
	Balapiravir	DENV	Lim et al., 2015 [61]	Balapiravir failed to be effective for patients with DENV. No efficacy was found in phase II Clinical trial.
	N-sulfonyl-anthranilic acid derivatives	DENV	Niyomrattanakit et al., 2010 [17]	NITD-29 inhibited DENV-2 in a virus titer reduction assay, neither NITD-1 nor NITD-2 exhibited any antiviral activity in cell culture.
	2’E-7D-ATP	DENV	Latour et al., 2010 [18]; De Burghgraeve et al. 2013 [64]	2’E-7D-ATP was able to inhibit all DENV serotype replication. However, the catalytic efficiency of incorporating this molecule was 10-fold lower than that of ATP.
	Modified tri-phenylmethyl nucleosides	DENV, YFV	Chatelain et al., 2013 [23]	The finding of these lipophilic structures should stimulate the interest for further structure activity research.
	2’,5’-bis-O-tritylated uridine	DENV, YFV	Chatelain et al., 2013 [23]	2’,5’-bis-O-tritylated uridine proved to be successful. This discovery was followed by synthesis of a series of close analogues, as a first attempt for setting up a structure activity relationship (SAR).
	3’,5’-bis-O-tritylated uridine analogues	DENV, YFV	Chatelain et al., 2013 [23]; Saudi et al., 2014a [25]	These simple lipophilic structures show strong YFV inhibiting properties, further structure activity research is warranted.
	Compound 27, 29i and 29	DENV	Lim et al., 2016 [11]	Compound 27 inhibted all four serotypes when inhibition studies were performed with clinical isolates of DENV1-4. Compound 29i was the next most potent compound and 29 displayed relatively lower cellular inhibition.
no speCIFIC ACTION	Lycorine	WNV, YFV, DENV	Zou et al., 2009 [21]; Lim et al., 2013a [38];	Lycorine potently inhibited flaviviruses in cell culture, it was reported to reduce viral titers of WNV, DENV, and YFV mainly through suppression of viral RNA replication.
	Amodiaquine	DENV	Boonyasuppayakorn et al., 2014 [33]	Amodiaquine inhibited DENV2 infectivity measured by plaque assay.
	AS101	WNV	Indenbaum et al., 2012 [69]	AS101 had a potential preventive and therapeutic effect against WNV infection.
	7-deaza-2’-C-acetylene-adenosine	DENV	De Burghgraeve et al., 2013 [64]	7-deaza-2’-C-acetylene-adenosine showed anti-DENV activity in cell culture and significantly reduced viremia in a mouse model.
	I45DC	DENV, YFV	Saudi et al., 2014b [70]	I45DC had a high dengue virus inhibitory activity.
	P23DCs derivatives	DENV, YFV	Saudi et al., 2014b [70]	P23DCs derivatives had potent DENV and YFV inhibitory properties.
	Favipiravir (T-705)	FYV, WNV	De Burghgraeve et al., 2013 [64]; Furuta et al., 2013 [71]	Lack of good in vitro antiviral activity of Favipiravir (T-705). Considerably higher concentrations of this compound were required.
	T-1105 and T-1106	FYV, WNV	Furuta et al., 2009 [72]	There are insufficient data to clarify the reason why the in vivo antiviral effect of T-1106 did not correspond to its in vitro activity and why there were such differences in antiviral activities between T-1106 and T-705. T-1106 can be a useful drug for YFV in humans.
	NITD008	ZIKV	Deng et al., 2016 [73]; Xie et al., 2016 [43]; Adcock et al., 2017 [27]	NITD008 showed potent anti-ZIKV activity. It could serve as a reference inhibitor for future drug screen and discovery.
	ZINC33683341 and ZINC49605556	ZIKV	Sandun et al., 2016 [74]	The results obtained from these assays are a starting point for drug discovery targeting Zika virus and other emerging pathogens.

The flavivirus genus contains more than 53 members, including yellow fever virus (YFV), dengue (DENV), West Nile (WNV), Japanese encephalitis (JEV), tick-borne encephalitis (TBEV) and Zika Virus (ZIKV) [3–7]. Members of this genus cause some of the most important diseases among emerging diseases; this is evident by an increased DENV prevalence in tropical and subtropical areas of the world, the emergence of WNV in North America, and the propagation of JEV in a large part of Asia and Oceania. Although authorized vaccines exist for yellow fever, Japanese encephalitis, and tick-borne encephalitis, these have had limited success in containing epidemics [8], while vaccines for other diseases caused by flaviviruses have yet to be developed [3]. Research on possible vaccine candidates for DENV, however, is currently being conducted; for example, in a recent phase III study, Villar et al., reported that a tetravalent vaccine (CYD-TDV) for dengue that has been assessed in children from five countries in Latin America, where dengue is endemic, is efficient against virologically confirmed dengue (VCD) [9, 10], and after several decades of efforts, the first vaccine was recently licensed for use, but confers only partial cross protection for the four DENV serotypes [11].

The flavivirus genome contains an open reading frame, flanked by 5’- and 3’- untranslated regions that presents a secondary structure essential for translation and replication [3]. This polyprotein cleaves into ten proteins (three structural proteins: C (capsid), prM (membrane protein precursor), and E (envelope protein); and seven nonstructural proteins: NS1, NS2a, NS2b, NS3, NS4a, NS4b, and NS5) [1, 12].

To date, diverse inhibitor compounds of several flavivirus proteins have been evaluated, mainly from proteins with enzyme function, such as NS3 and NS5, and some molecules have been patented for this purpose, as well as to treat, prevent and alleviate infections caused by WNV, DENV and JEV [13, 14] On the other hand, peptides blocking virion envelope protein binding to host cell membranes have been designed [15], and sequences for protease substrates of DENV and WNV have been proposed [16].

In dengue, for example, the use of N-sulfonylanthranilic acid derivatives has been described in allosteric inhibition of the NS5 protein [17], while beta-d-2’-ethynyl-7-deaza-adenosine triphosphate (2’E-7D-ATP) has been used in competition with the natural nucleotide [18]. Furthermore, compounds with no specific target have also been reported, which have been shown to affect the viral replication cycle, such as curcumin, a natural compound with several inhibitory effects in in vitro dengue type 2 virus-infected cells [19]. However, until now no antivirals compounds have been approved to treat diseases caused by flaviviruses [11].

This article highlights the main characteristics of flaviviruses, their importance in the search for inhibitor molecules, and the development of compounds that, to date, have been tested for the alteration of the replicative process of viruses of the genus.

## Viruses of the flavivirus genus

The flavivirus genus contains several important human pathogens, including tick-borne encephalitis virus, Japanese encephalitis virus, West Nile virus, dengue virus, yellow fever virus and Zika virus [3, 4, 8, 20–25]. Most flaviviruses are transmitted to humans by arthropods; these types of viruses cause most of the emerging diseases. Currently, there are only three vaccines authorized against infections by flaviviruses: yellow fever virus, Japanese encephalitis virus, and tick-borne encephalitis virus [3, 8, 21] Annually, nearly 400 million cases of dengue are reported worldwide [26]. In the Americas, approximately 150,000 suspected Zika cases have been reported and more than 3000 cases have been confirmed since March 2014 through May 2016 [27]. All flaviviruses are antigenically related, as shown in hemagglutination inhibition assays with polyclonal serum antibodies [22].

## Flavivirus genome

Flaviviruses exhibit a spherical mature virion 500 A° in diameter, the genome is packaged by a capsid protein (C) into a host-derived lipid bilayer with 180 copies of embedded envelope protein (E). The E protein forms a complex with the membrane protein precursor (prM) during virion assembly in the endoplasmic reticulum. It forms immature particles transported to the Golgi compartment, where a cellular serine protease matures them, furin, which mediates the cleavage of prM to M, resulting in the homodimerization of the E protein into mature fused competent particles before going into circulation. The flavivirus genome spans approximately 11 Kb; it is composed of single-stranded positive-sense RNA and contains a single open-reading frame flanked by two 5’- and 3’- untranslated regions with secondary structures essential for translation and replication initiation [1, 3, 21].

## Flavivirus polyprotein

The virus enters the host cell through receptor-mediated endocytosis [28–31] and endosome acidification [32]. The flavivirus genome is released into the cytoplasm immediately after cell entry (including monocytes, macrophages, and dendritic cells) [3] by viral glycoprotein-mediated membrane fusion [31]. The genome codes for a single polyprotein processed through proteolysis to produce three structural proteins: (E, prM, and C) and seven nonstructural proteins: (NS) (NS1, NS2a, NS2b, NS3, NS4a, NS4b, and NS5) [3, 31, 33]. The polyprotein is co- and post-translationally cleaved by action of a combination of furin-type or other cellular proteases located in the Golgi compartment, as well as by the viral serine protease embedded in the N-terminal domain of the NS3 protein (NS3 Pro), which requires NS2b for its activity [3]. It is possible that NS proteins undergo a co-translational assembly in endoplasmic reticulum membranes, forming the competent replication complex, and later differ regarding the function and composition of nonstructural proteins [2].

## Flavivirus structural proteins

The C protein, approximately 11 KDa, interacts with the viral genomic RNA, forming the nucleocapsid (NC), a functional region required for dimerization in viral assembly. The capsid protein contains an internal hydrophobic sequence that mediates membrane association [3].

The glycosylated prM, 26 KDa, is processed from the polyprotein in the endoplasmic reticulum by host signaling, through divisions in the N-terminal site of the capsid-prM and prM C-terminal-E protein associations. The association of prM-E produces immature non-infectious viral particles; these immature particles travel through the Golgi apparatus and a reversible morphological change of the E protein is produced before prM processing [3, 22].

Cleavage of prM to M (membrane protein) by a cellular serine protease, furin, produces an irreversible conformational change in E. The peptide cleaved from prM is retained in the virion and released after the virion has segregated and is exposed to a neutral pH, thus, protecting the E protein from premature fusion [3, 22].

The E protein, 53 KDa, in its dimer form, is the main component of the virion surface; in this form, the E protein is competent for cell surface binding, fusion, and viral entry into the host cells [3, 34]. The amino acid sequence identity of the E protein is at least 40 to 44% among flaviviruses [22].

## Flavivirus nonstructural proteins

Nonstructural proteins are essential for virus replication, virion assembly, and invasion from the host’s immune response; these are located mainly in the cytoplasm, forming replication complexes involved in viral RNA synthesis [35].

The NS1 protein has a molecular weight of 46–55 KDa, depending on its state of glycosylation. It is present in multiple oligomeric forms, and is found in different cellular locations. Intracellular NS1 plays an essential role in virus replication and has proven to co-locate with dsRNA and other components of the replication complex; moreover, it can induce complement-mediated inhibition of the immune response and has the capacity to remodel membrane lipids [31]. Nonetheless, its precise protein function has not been elucidated, yet, it is known that the protein is highly immunogenic and can be proposed as the component of a new vaccine against flaviviruses [22, 36].

Regarding the NS2A protein, it has no enzymatic function, but it is necessary for virus replication and assembly [31, 35].

The NS2B protein is an integral membrane protein of 14 KDa that contains three hydrophobic domains: two transmembrane segments located at the N- and C-terminal ends and a central region of 47 amino acids (from residue 46 to 97), which acts as an essential cofactor for protein NS3) [3, 37].

NS2B binds to NS3 in the membrane, which is required for viral replication complex maturation [37].

The NS3 protein, 69 KDa, is the second longest viral protein in the flavivirus genome, after NS5, and plays an essential role in the viral replication cycle. NS3 contains a protease domain in the N-terminal region that cleaves the viral protein precursor into individual NS proteins, and a C-terminal RNA helicase/NTPase domain, which participates in genome replication and viral RNA synthesis. In order to function as an active enzyme, the NS3 protease requires NS2B as a cofactor [31, 37].

The NS3 flavivirus protein is not soluble or catalytically active as an in vitro protease, suggesting that it does not fold properly without the NS2B protein. The helicase activity of NS3 is believed necessary for fusion of secondary structures before initiation of RNA synthesis or for the RNA duplex, whether to separate dsRNA compounds formed during viral RNA synthesis or as a translocase that can eliminate proteins bound to the viral RNA [37].

The NS4B protein (approximately 27 KDa) is an integral membrane protein with high hydrophobicity and moderate conservation with approximately 40% amino acid similarity among flaviviruses [35]. The NS4A protein induces membrane reordering and autophagy to improve viral replication, while NS4B modulates the host’s immune response by suppressing interferon α/β signaling and NS3 helicase activity [31]. These two proteins do not show an identified enzymatic activity, but are believed to serve as essential scaffolds for viral replication complex formation, along with NS2A and NS2B [35, 38].

The NS5 protein (close to 100 KDa) is the biggest viral protein coded by flaviviruses and the most highly conserved [2, 5], with a shared sequence identity of over 75% amongst all DENV serotypes [3]. The C-terminal domain structure of WNV and DENV NS5 proteins revealed that it is a classic polymerase [5]. NS5 is of great importance to viral replication, given that it performs two independent enzymatic activities separated by an interdomain region: an S-adenosyl methyltransferase (SAM) and an RdRp [3, 30, 39], essential activities for the viral replication cycle [40]. In addition, it codes the main type I interferon (IFN)-signaling antagonist [37, 41].

The RdRp of the NS5 protein initiates RNA synthesis through a *de novo* mechanism [5, 8] that differs from the primer-dependent mechanism used by other viruses. Like all polymerases, the structure of the flavivirus RdRp is similar to a right hand with characteristic fingers, palm, and thumb subdomains [5, 42].

## Development of flavivirus inhibitors

Identification of small molecules that inhibit a critical step in the viral cycle requires detailed biochemical and structural characterization of each protein essential for replication. The C, prM, and E viral proteins undergo conformational changes during viral particle entry, assembly, and exit; these changes can be targets inhibited by antiviral drugs [3].

In the case of dengue, development of an antiviral drug is complicated due to the presence of four serotypes; thus, while lifelong protection against one serotype is induced, protection against the other serotypes lasts only a few months [22]. Incomplete protection against a serotype can affect the outcome of the disease upon establishing infection by a different serotype, through a process known as antibody-mediated disease improvement, immunopotentiation, or antibody-mediated immunofacilitation.

Continuous interest exists for vaccines and drugs effective against viruses, such as dengue and West Nile viruses, due to the difficulties seen with existing drugs, such as the reported controversial effect of ribavirin on flaviviruses [2]. Besides, the recent interest in the search for inhibitors has also increased, due to the recent epidemics caused by the Zika virus, thus this disease has become a public health problem [43].

## Compounds used as flavivirus inhibitors

Diverse biological and synthetic compounds have been designed and evaluated in vitro and in vivo, including bioinformatics-based assays, seeking to induce inhibition of the most important protein activities in the viral replication process of this genus (see Table 1). Some of these compounds, however, have been tested with no specific target other than affecting viral particle replication. The compounds discussed ahead have been tested as flavivirus inhibitors and inhibitors of the viral cycle proteins of highest interest.

## Inhibitors of the E protein

In DENV, a few heterocyclic compounds, like NITD-448, compound 6 (with a quinazoline nucleus) [38], P02, D02, D04, D05, A5, and 1662G07 have been identified *in silico*. Some of these (for example, NITD-448) inhibit E protein-mediated membrane fusion, such as DENV-2 infection in cell culture. P02 inhibits viral replication at micromolar concentrations; as a result, it is considered a promising compound for future development of an effective treatment against dengue virus and related flaviviruses [44]. NITD refers to compounds developed by the Novartis Institute for Tropical Diseases, whose mission is to develop drugs against dengue [38].

Other compounds, like compound 6, present inhibition in clinical DENV isolates. D02, D04 and D05 inhibit the virus life cycle at steps other than replication, consistent with inhibition of maturation or virus entry into cells as a result of binding E protein. Further, P02 showed inhibition of YFV, and A5 against WNV, DENV, and YFV [38].

Moreover, various studies have shown that castanospermine and celgosivir also inhibit dengue virus replication, interrupting E protein folding, as well as prM and NS1 folding [38].

Reports indicate that the mimetic peptide DN59 [45], which matches with a region of the dengue virus envelope protein, inhibits the four dengue virus serotypes, as well as other flaviviruses. This compound strongly interacts with synthetic lipid vesicles and causes membrane alterations; yet, it was not toxic to mammal and insect cells. As a result, DN59 inhibits flavivirus infection through direct interaction with viral particles, resulting in genomic RNA release [46].

## Capsid inhibitors

Compound ST-148 is a DENV capsid inhibitor; this compound inhibits DENV with a titer reduction at EC50 of 0.016 μM [38] in addition to blocking the cytopathic effect caused by DENV. This compound is potent against the four dengue virus serotypes, as well as against other flaviviruses; its inhibitor effect was evaluated in in vitro and in vivo assays in mice (AG129), revealing that it significantly reduces viremia and viral loads in vital organs [47].

## NS3 protein inhibitors

Considering that the NS3 protein performs various important processes in viral replication, diverse inhibitors have been designed against its protease and helicase activities; thus, it is a promising protein for vaccine and treatment development for flavivirus diseases.

## Protease domain inhibitors

Diverse inhibitors of the flavivirus protease domain have been evaluated; for example, NS3 has been proposed to interact with the Bowman-Birk-type polypeptide inhibitor, isolated from mung beans, and represents the only structure of a DENV2 NS3 complex available to date. This bivalent inhibitor contains a lysine and arginine occupying the substrate-binding S1 pocket; therefore, it affects NS3 binding to NS2B by binding to this pocket [2].

The flexible behavior of the DENV NS3 protease, as seen through computational tools, is not observed with three ligand complexes of WNV, all containing the NS2B cofactor. One of them harbors aprotinin as an inhibitor ligand, while the other two form a complex with an analogous peptide-type substrate covalently bound to a residue of the catalytic triad Ser135. Aprotinin shows the highest specificity to the NS3 protease pocket [2].

Compound BP13944, a quaternary ammonium salt, is identified as a dengue protease inhibitor, through high-throughput screening (HTS) of 60,000 chemical compounds, using a BHK-21 (Baby Hamster Kidney fibroblasts) stable cell line containing an efficient DENV-2 luciferase replicon. BP13944 reduced DENV reporter replicon expression in cells, showing a half-maximal effective concentration (EC50) of 1.03 ± 0.09 μM. With no detectable toxicity, the compound inhibits viral RNA replication or synthesis in all dengue serotypes, although not in the JEV [48].

Additionally, keto amides have been evaluated as dengue virus protease inhibitors in cell culture, with reports that compound 32 inhibits replication in a dose-dependent fashion, reducing viral titers 1000-fold to non-cytotoxic concentrations, determined through luciferase measurement as a cell viability marker [49].

A set of 45 inhibitor peptides was designed, synthesized, and evaluated against the dengue virus NS2B-NS3 protease, finding that the uncharged WCW-NH2 tetrapeptide inhibits protease in the four serotypes [50].

Furthermore, anthracene-based compounds, ARDP0006 and ARDP0009, have been identified through virtual screening of a chemical compounds library by identifying small molecules that fit in defined target sites, such as the active site and P1 pocket of the DENV protease, finding that these compounds inhibit dengue-2 virus replication in cell culture [51]. ARDP0006 (anthraquinone) was the most potent inhibitor among all others evaluated, which reduced viral titers more than 1 log PFU/mL in HuH-7 and K562 cell lines [52].

Currently, few compounds show adequate properties for drug development, which may be attributed to the fact that inhibitors weakly bind to the enzyme, given that classic serine-protease inhibitors are inefficient or have a low potential against the DENV NS3 protease. One exception, though, is aprotinin, which envelops the enzyme and keeps the substrate from accessing the protease active site [53].

## Helicase domain inhibitors

The crystalline structure of the DENV NS3 helicase domain reveals that this domain does not contain any pockets for possible binding to small inhibitor molecules [53, 54].

Despite scarce reports of NS3 helicase inhibitors, to date, the existence of halogenated benztrioles that inhibit the WNV helicase has been reported [3], and ivermectin, an antiparasitic drug against helminths, has been reported to display inhibition against the helicase activity of DENV, JEV and YFV [10, 55–57].

Recently, there have also been reports that ST-610 (discovered in assays monitoring the cytopathic effect of DENV) acts as a helicase inhibitor, selectively inhibiting the four DENV serotypes in cell culture, but it does not inhibit the ATPase activity [38, 57].

Moreover, suramin (a polysulfonated compound used as an antihelminthic) has been shown to act as a non-competitive inhibitor of the DENV helicase; this compound was discovered for DENV inhibition through screening of a compound library using a molecular probe-based helicase assay [57, 58].

Similarly, analogues of the ML283 compound (a benzothiazol oligomer derived from the primuline dye), which inhibit the hepatitis C virus (HCV) helicase, are active against DENV replicons, acting as potent inhibitors of NS3 through catalysis by ATP hydrolysis. In addition, pyrrolone specifically inhibits NS3 capacity to cleave ATP, as well as 50% of DENV and WNV (subtype Kunjin) replication in cells [57].

Finally, helicase inhibitors have been shown to serve as antiviral therapy against flaviviruses and this was recently reviewed [37, 38].

## NS4 protein inhibitors

The NS4 protein, although not having a specific enzymatic activity, is also an important target against flaviviruses. Recently, a new dengue virus inhibitor against the NS4B viral protein was identified, which restricts genomic RNA replication instead of viral genome translation; nevertheless, further studies are required to elucidate the mechanisms responsible for the antiviral properties of SDM25N (a δ-opioid receptor antagonist). SDM25N was extracted from a small molecule library and exhibited antiviral activity in Hela and BHK-21 cell lines, although not in C6/C36 cell lines derived from the *Aedes albopictus* mosquito [59].

Similarly, NITD-618 has been identified as an NS4B inhibitor, selected from a library of almost 1.8 million small molecules, this compound was found to be active against the four DENV serotypes, but inactive against other RNA viruses, including two other flaviviruses like WNV and YFV [38].

## NS5 protein inhibitors

As mentioned, the NS5 protein displays a fundamental activity for the development of the flavivirus replication cycle, making this protein an important therapeutic target [3, 38, 40, 60, 61] that can be attacked to affect viral RNA production. The inhibitory compounds discussed ahead have been evaluated against the two activities of this viral protein.

## Methyltransferase domain inhibitors

Reports exist on the use of sinefungin, a SAM (S-adenosyl-L-methionine) analogue, as a broad-spectrum DENV inhibitor, Wesselsbron, and WNV methyltransferases. Sinefungin contains a carbon and amine instead of the methylated sulfur present in SAM, and the amine imitates the charged sulfur. The ligand binds to the SAM site in a similar way as SAM, but does not undergo similar interactions with the protein; despite this, interaction assays with DENV MTase show that sinefungin affinity is six times greater than SAM affinity [61].

Furthermore, other DENV NS5 protein inhibitors have been identified, such as S-adenosyl homocysteine (SAH) and Compound 10 (as inhibitors of the methyltransferase active site), guanosine monophosphate (GMP), BG-323, and aurintricarboxylic acid (with binding sites to the guanosine triphosphate (GTP) pocket), and 4-HPR (fenretinide) and ivermectin with unknown binding sites [38, 61].

S-adenosyl homocysteine, compound 10, GMP, and sinefungin did not show good progress because these are non-cell-permeable compounds. On the other hand, no information is available regarding BG-323 and aurintricarboxylic acid; while 4-HPR has shown to be efficient in a mouse model lacking type I and II IFN receptors and ivermectin is undergoing phase II/III clinical trials [61].

Ribavirin, a synthetic guanosine analogue, has shown to inhibit dengue methyltransferase and hepatitis C virus replication [51, 62].

An assay with Rhesus monkeys showed that monkeys treated with ribavirin, as well as those treated with the placebo, developed viremia, with a peak between days 3 and 9 post-infection. In addition, there were no significant differences in the time of infection onset, duration, or viremia level in the two groups evaluated. Thus, ribavirin resulted inefficient as a prophylactic drug for type-1 dengue virus infection [63].

## RNA-dependent RNA polymerase domain inhibitors

Flavivirus RdRp have been considered among the most interesting drug targets because viral polymerase activity is essential for replication and human host cells lack this enzyme [8, 64, 65]. RNA polymerase inhibitors are divided into two classes: nucleosides inhibitors (NI) and non-nucleosides inhibitors (NNI) [8, 17, 38, 53, 61, 66]. Nucleoside analogues are generally converted into nucleosides by host cell kinases [8]. Recently, two NNI: N-(4-hydroxyphenyl) retinamide and ivermectin have been identified in binding assays as compounds able to block NS5 [61].

Ivermectin has been reported as an inhibitor of importin α/β and, consequently, of the NS5 polymerase, given that the former is required for its activity. Prior treatment with ivermectin inhibits dengue virus infection in vero cells; additionally, pre-treatment with this compound has been found to strongly inhibit the nuclear localization of NS5 during DENV-1 and DENV-2 infection in BHK-21 or Huh-7 cells [56]. However, problems related to the toxicity of ivermectin may present significant hurdles for its potential use in anti-DENV therapy [61].

On the other hand, the viral polymerase inhibitor 7-deaza-2’-C-methyladenosine (7DMA) was identified as a potent ZIKV inhibitor. A mouse model for ZIKV infections, which was validated for antiviral studies, demonstrated that 7DMA markedly delays virus-induced disease in this model [67].

Nucleotide/nucleoside analogues induce termination of the nucleic acid chain or act as natural substrates through competition due to their broad-spectrum antiviral activity, or can cause mutation by incorporation to a newly synthesized RNA chain [8, 42]. Also, NNI allosterically bind to pockets and/or surface cavities, thus, blocking enzyme activity; this mechanism includes structural alteration of the polymerase to an inactive conformation, blocking the conformation required for elongation initiation or preventing polymerase elongation [8, 17].

To date, few molecules, like flavivirus RdRp NIs and GTP analogues 3’dGTP, ddGTP, 3’dioxolane 3’dGTP, and 2’-O-methyl GTP have shown low IC50 values in in vitro activity assays with DENV RdRp.

There is an alleged NNI, ammonium-21-tungsto-9-antimoniate, also called HPA 23, which inhibits DENV2 RdRp; this NNI was proposed to inhibit the HIV reverse transcriptase through competition with the nucleic acid template, resulting in a non-allosteric-type inhibitor [8].

Moreover, NITD 008 (an adenosine nucleoside) [38, 68], NITD 203 (a related compound) [30], and balapiravir (RG1626) (a cytidine nucleoside) have been reported as RdRp domain inhibitors [38, 61]. Both NITD 008 and NITD 203 induced inhibition of the four DENV serotypes, as well as of YFV and WNV [30]. NITD 203 proved efficient in in vitro and in vivo studies against dengue; however, it did not satisfactorily achieve non-observable levels of adverse effects within 2 weeks in in vivo studies [68]. Additionally, in vitro studies showed that NITD 008 activation was less effective in dengue pre-infection, compared to other compounds, although preclinical studies have shown that this drug presents toxicity problems [61].

In addition, balapiravir does not show efficiency in phase II clinical studies, given that this drug, initially developed for hepatitis C treatment, it failed to achieve efficacy in dengue patients [61].

In contrast, dengue virus polymerase enzymatic activity has been inhibited allosterically through blockage of the RNA tunnel by using N-sulfonylanthranilic acid derivatives, considered desirable to develop antiviral compounds [17]. Furthermore, the activity of this enzyme has been inhibited by action of beta-d-2’-ethynyl-7-deaza-adenosine triphosphate (2’E-7D-ATP) through competition with the natural nucleotide. This nucleoside analogue, initially developed for HCV, displayed anti-dengue activity in cell culture and significantly reduced viremia in a mouse model with DENV; nevertheless, the catalytic incorporation efficiency of this molecule is tenfold less than that of adenosine triphosphate (ATP) [18, 64]. Similarly, a study on the functional analysis of two cavities of the flavivirus NS5 polymerase determined that the B cavity could be a target for drug design because amino acid residues Leu 328, Lys 330, Trp 859, and Ile 863 are essential for viral replication [5].

Additionally, various modified triphenylmethyl nucleosides have shown in vitro anti-YFV and anti-DENV activity, although the antiviral activity of several of these compounds (4b, 4c, and 5b) was weaker in DENV [23].

2’,5’-bis-O-tritylated uridine has been identified as a flavivirus inhibitor, specifically for YFV, in addition to some synthetic nucleoside analogues with different tritium regioisomers, which have been evaluated to establish a structure-activity relation (SAR) [23]. The compound inhibited YFV and DENV replication in a low μM range, proving that it can potentially inhibit the RdRp [64]. Other in vitro studies have reported the activity of 3’,5’-bis-O-tritylated uridine analogues for DENV and YFV RNA polymerase inhibition [23, 25].

Recently, the characterization of a novel allosteric pocket at the interface of the thumb and palm subdomains of DENV RdRp has been reported. It is located near the priming loop (aa782-809) of the enzyme and is lined by highly conserved residues across DENV1-4, as well as in other flaviviruses, including ZIKV. Inhibitors generated by rational design potently inhibited DENV1-4 polymerase *de novo* initiation activities and virus replication in various cell types. They bind with strong affinity to recombinant apo-enzyme, as well as FL NS5 from DENV replicon cell lysates. Compound 29 was one of the most potent compounds in the series, and compounds 27 and 29 were inactive when tested with the WNV replicon cell-based assay [11].

## Inhibitors with non-specific action

Lycorine, which has a reported antiviral activity, has been evaluated against a number of flaviviruses and has acted as a WNV, YFV and DENV-1 inhibitor [21, 38], although it has no WNV protease, NTPase, MTase, or RdRp inhibitor activity. Therefore, its action is not addressed directly against the enzymatic functions of the viral proteins [21].

Assays with amodiaquine (an anti-malaria drug) have shown that this compound presents activity against DENV-2 and inhibits RNA replication and virus infectivity in BHK-21 cells; however, it has been determined that neither the protease nor the polymerase are probable targets of this compound [33].

The action of AS101 (a non-toxic immunomodulator) has also been evaluated, showing anti-WNV effects in in vitro and in vivo systems [69].

An adenosine analogue (7-deaza-2’-C-acetylene-adenosine) has functioned as a potential inhibitor of DENV replication in cell culture and in a mouse model; however, this compound has shown serious effects in in vivo toxicity studies on rats and dogs [64].

In addition, DENV and YFV inhibition has been reported for derivatives of imidazole 4,5 dicarboxamide (I45DC), and pyrazine 2,3 dicarboxamide (P23DCs) [70].

Favipiravir, T-705, a pyrazine-substitute compound used in clinical trials to treat human influenza virus infections [64], inhibits various viral pathogens, including YFV and WNV, although greater concentrations are required for the latter two viruses in in vitro assays and in rodents, compared to influenza virus. It is worth noting that no envelope protein was reportedly found in WNV in treated animals 7 days after virus exposure; furthermore, oral administration of favipiravir in hamsters infected with YFV, starting four hours prior to virus exposure, protected the animals from death [71]. T-705 has also significantly improved survival and disease parameters in YFV-infected hamsters, despite the fact that this compound lacks good activity in in vitro assays [64].

Further studies are needed to evaluate T-705 as an antiviral treatment for WNV, given that it is one of the few known compounds that reduce mortality by WNV in rodents [72].

Studies on the action mechanism of T-705 have shown that the compound is converted into a triphosphate ribofuranosyl derivative by host enzymes and that it selectively inhibits the influenza virus RNA-dependent RNA polymerase, with no toxicity in mammal cells [72].

Furthermore, T-705, T-1105, and T-1106 derivatives can be considered candidates because these have shown good activity in treatments in laboratory animals infected with RNA viruses, including WNV and YFV. T-1106 showed surprising therapeutic efficacy in a hamster model of YFV In any case, it is expected that T-1106 will be a useful drug for YF in humans [72].

On the other hand, it has been indicated that NITD008 (an adenosine analog) is an effective antiviral compound that protects mice from lethal ZIKV challenge. This study demonstrates that the A129 mouse model could be used for testing the in vivo efficacy of ZIKV inhibitors [27, 43, 73].

Also, has been report the susceptibility of Zika virus envelope protein (ZVEP) to inhibition via two small molecules, ZINC33683341 and ZINC49605556 by preferentially binding onto the primary receptor responsable for the virus’ virulence. Antiviral activity was confirmed when ZINC33683341 was tested in cell culture [74].

## Conclusions

Flaviviruses cause some of the most important diseases among emerging diseases, leading to a much-needed constant search for inhibitory compounds, given that only a few of these viruses currently have an approved vaccine, while others remain under evaluation in the search for substrates that affect the viral replication process, because until now no antiviral compounds have been approved to treat diseases caused by flaviviruses. Due to their endemic nature, JEV, WNV, DENV, FEV, TEBV and ZIKV are among the viruses of greatest interest [3, 6]. NS3 and NS5 are among the most important protein targets because they carry out enzymatic functions of high interest [3, 38] and are part of the replication complex, along with viral RNA, viral cofactors and host cell cofactors [37], making them attractive inhibitor targets [31, 37, 40].

Aprotinin and anthracene-based compounds are among the molecules used to affect DENV NS3 protein activity by affecting the protease domain [53], in addition to ivermectin, suramin, and pyrrolone, among others, with action on the helicase domain [55–57]. As for the RdRp, an important role is played by nucleoside and non-nucleoside-type inhibitors, including GTP and adenosine analogues (NITD 008 and NITD 203) [30], and 2’E-7D-ATP [18, 64], as well as other molecules that induce DENV inhibition by allosteric coupling.

This review emphasized the different inhibitor compounds evaluated to affect replication of flaviviruses related to the most endemic diseases, like DENV, providing a broader vision of the types of molecules tested to date and of others that could be evaluated to achieve the final objective of many researchers throughout the world interested in studying flaviviruses: to find one or more compounds with the possibility of being vaccine candidates or that can be used as treatments for diverse effects produced by the presence of these viruses in the organism.

## Abbreviations

2’E-7D-ATP: Beta-d-2’-ethynyl-7-deaza-adenosine triphosphate
ATP: *Adenosine triphosphate*
BHK-21: Baby Hamster Kidney fibroblasts
C: Capsid protein
CYD-TDV: Tetravalent Dengue Vaccine
DENV: Dengue virus
E: Envelope protein
EC50: Half-maximal effective concentration
GMP: Guanosine monophosphate
GTP: Guanosine triphosphate
HCV: Hepatitis C virus
HTS: High-throughput screening
JEV: Japanese Encephalitis Virus
M: Membrane protein
Mtase: Methyltransferase
NC: Nucleocapsid
NI: Nucleosides *inhibitors*
NITD: Novartis Institute for Tropical Diseases
NNI: Non-nucleosides *inhibitors*
NS: Nonstructural proteins
PFU: *Plaque-forming unit*
prM: Membrane protein precursor
RdRp: RNA-dependent RNA polymerase
SAH: S-adenosyl homocysteine
SAM: S-adenosyl methyltransferase
TBEV: Tick-Borne Encephalitis Virus
VCD: Virologically confirmed dengue
WNV: West Nile virus
YFV: Yellow fever virus
ZIKV: Zika virus
